# Beam shaping in high-power broad-area quantum cascade lasers using optical feedback

**DOI:** 10.1038/srep44284

**Published:** 2017-03-13

**Authors:** Simon Ferré, Louise Jumpertz, Mathieu Carras, Robson Ferreira, Frédéric Grillot

**Affiliations:** 1 Thales Research & Technology, 1 avenue Augustin Fresnel, 91767 Palaiseau, France; 2 MirSense, Centre d’intégration NanoINNOV, 8 avenue de la Vauve, 91120 Palaiseau, France; 3 Laboratoire Pierre Aigrain, Ecole Normale Supérieure-PSL Research University, CNRS, Université Pierre et Marie Curie-Sorbonne Universités, Université Paris Diderot-Sorbonne Paris Cité, 24 rue Lhomond, 75231 Paris Cedex 05, France; 4 Université Paris-Saclay, Télécom ParisTech, 46 rue Barrault, 75013, Paris, France; 5 Center for High Technology Materials, University of New-Mexico, 1313 Goddard SE, Albuquerque, NM, United States

## Abstract

Broad-area quantum cascade lasers with high output powers are highly desirable sources for various applications including infrared countermeasures. However, such structures suffer from strongly deteriorated beam quality due to multimode behavior, diffraction of light and self-focusing. Quantum cascade lasers presenting high performances in terms of power and heat-load dissipation are reported and their response to a nonlinear control based on optical feedback is studied. Applying optical feedback enables to efficiently tailor its near-field beam profile. The different cavity modes are sequentially excited by shifting the feedback mirror angle. Further control of the near-field profile is demonstrated using spatial filtering. The impact of an inhomogeneous gain as well as the influence of the cavity width are investigated. Compared to existing technologies, that are complex and costly, beam shaping with optical feedback is a more flexible solution to obtain high-quality mid-infrared sources.

Quantum Cascade Lasers (QCLs) have undergone perpetual research efforts for the last two decades, making them the most appealing coherent light source in the mid-infrared range. In fact, they have been proved to be a stable, easily integrable, robust, efficient and powerful laser source operating at room temperature^1^. They are suiting many application needs, either in the civil domain, with chemical species spectroscopy and open space telecommunications for example, or military and defense fields, with explosive and drug detection. In order to address more demanding applications, such as very high precision spectroscopy, selective surgery or infrared countermeasures, QCLs with even higher power and luminance are required. A straightforward idea to increase the power of a laser diode is to enlarge the active region, and especially the laser width. Hence, QCLs as broad as 400 μm have shown record-breaking output peak powers as high as 120 W^2^.

However, such devices are strongly affected by both thermal and optical issues hence showing rather poor beam quality performance. Indeed, even if the thermal resistance decreases with the ridge width, the thermal load becomes too important to be dissipated efficiently. The laser therefore needs to be operated with very short pulses to avoid thermally degraded performances, or even device destruction, which limits the mean optical power. Furthermore, a larger cavity will support numerous transverse modes, the lasing transverse mode is no longer the fundamental mode and the far-field pattern is typically bi-lobe. Several solutions have been proposed to improve the beam quality of broad area (BA) devices. QCLs with photonic cristals (PC) etched on top of the active region with diffraction-limited single-lobe far-field have been studied^3^ and reported at 4.36 μm^4^, 4.75 μm^5^ and 7.8 μm^6^. Moreover, architectures with a tilted facet have shown an improved far-field^7^,^8^,^9^. Likewise, even if they present a smaller gain region compared to BA QCLs, tapered QCLs are an interesting trade-off between large effective active region, high power, and good beam quality^10^,^11^. Another approach to solve both thermal and optical drawbacks of BA QCLs is to split the ridge into an array of micro-stripes optically coupled to each other to achieve a stable optical supermode. The far-field is typically two-lobed in the case of evanescent coupling^12^,^13^, but single-lobe emission has been achieved using stripe antiguided laser arrays^14^.

Nevertheless, all these solutions require monolithic integration, and are therefore highly depending on the fabrication steps repeatability and quality. These technologies lack flexibility and require costly additional processes such as electronic lithography or semiconductor regrowth.

In interband semiconductor BA lasers, inducing external perturbations such as optical feedback or optical injection is an efficient technique to control the beam quality and dynamical stability, without resorting to integrated solutions^15^,^16^,^17^,^18^. For instance, applying optical feedback enhances the beam quality by reducing substantially the filamentation, which is one of the main issues of BA laser diodes. Filamentation corresponds to fast spatio-temporal oscillations, due to diffraction of light, self-focusing and spatial hole-burning, whose position along the laser cavity fluctuates with time. Even though the origin of filamentation is not fully understood yet, it leads to the excitation of higher spatial modes, with different phase velocities, hence deteriorating significantly the laser coherence and the beam quality. Moreover, the appearance of filamentation in a BA laser is related to the linewidth enhancement factor (LEF) value of the device^19^. This parameter quantifying the coupling between amplitude and phase of the electric field in semiconductor lasers has an impact on many key properties of the lasers, such as its optical linewidth or its dynamical stability. The higher the LEF, the more the laser beam quality will be impacted by filamentation. Optical feedback can be used to counter the filamentation-induced drawbacks, without altering other performance of the laser. Furthermore, the dynamics ruling a BA semiconductor laser are complex, originating from the competition between the many transverse modes that coexist in the cavity. Strong instabilities or even chaos may appear in the emitted signal of a free-running BA laser diode, which can also be compensated using optical feedback.

The impact of optical feedback on the near-field profile and dynamical behavior of a BA semiconductor laser is ruled by three main parameters. The first one is the sign of the population-inversion induced index change, i.e. whether the laser design is based on gain-guiding (positive index variation) or index-guiding (negative index variation). Studies have shown that total stabilization of the emission pattern, where optical feedback forces the laser to operate on the fundamental transverse mode, can only be achieved in the case of negative population-induced index change^20^. Furthermore, the two other key parameters are the feedback strength, defined as the ratio between reinjected and emitted powers, and the external cavity length. Depending on these parameters, higher spatial modes will be either excited or suppressed^17^,^18^. As the number of excited modes increases, the dynamical behavior will switch from stable emission, to pulse package fluctuations and finally to fully developed chaotic state^16^.

Spatially filtered optical feedback can further improve the near-field profile of the laser emission. Reinjecting only the central part of the emitted beam will indeed favor the lower order modes, leading to a high quality beam profile close to the single-transverse mode behavior^18^.

Compared to interband lasers, QCLs have a low LEF, hence reducing the risk for filamentation. However, applying optical feedback could improve the beam quality of BA QCLs and make them suitable sources for high power mid-infrared applications. Recent studies have indeed shown that optical feedback has a similar effect on narrow-ridge QCLs than on their interband counterparts, resulting in single- or multimode evolution of the optical spectrum^21^ and even sometimes to the occurrence of chaotic dynamics in the structure^22^.

In this work, conventional and spatially-filtered optical feedback will be applied to a 32 μm -wide QCL. The high performances of this QCL emitting around 4.6 μm is first detailed. In particular, we will report that this laser presents high mean and peak powers, efficient heat dissipation allowing operation at high duty cycle, as well as high quality far-field over the whole range of operation. In a second part, the impact of optical feedback on the laser near-field is studied as a function of the feedback mirror angle, showing significant modifications on the near-field pattern. Strong regeneration of the profile is achieved in the case of centered feedback using spatial filtering. Furthermore, the response of a QCL with poor far-field quality to feedback is investigated, as well as the influence of the laser ridge width by comparing to a 14 μm -wide device.

## Results and Discussion

### Laser performances

**Figure 1:**
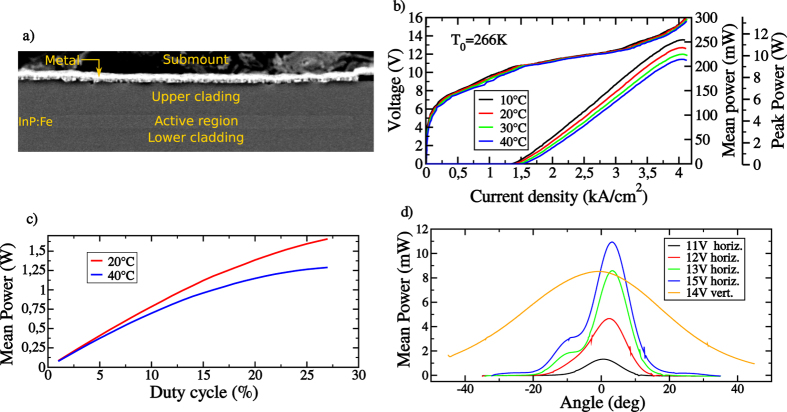
Laser performances. (**a**) SEM picture of the device facet mounted epi-side down. The 32 μm active region (the lighter area) is surrounded by InP:Fe and sandwiched between two n-doped InP cladding layers.(**b**) PI and IV curves at 3% duty cycle for temperatures from 10 °C to 40 °C. (**c**) Evolution of the mean power with duty cycle at 20 °C and 40 °C. (**d**) Far-fields at different (I, V) horizontal and vertical.

The studied QCL is 32 μm -wide, 4 mm -long, gold high-reflection (HR)-coated on the back facet and mounted epi-side down onto an AlN submount. The mounted device is shown on Fig. 1a.

Standard voltage vs current and power vs current (PIV) curves are measured at temperatures ranging from 10 °C to 40 °C. Results are shown in Fig. 1b. The threshold current density is 1.51 kA/cm^2^ at 10 °C, and 1.69 kA/cm^2^ at 40 °C, which yields a characteristic temperature of *T*_0_ = 266 *K* in the 10 °C–40 °C temperature range, which is in accordance with previously published results for QCLs with the same design^23^. The low values of the current densities show that the current leakage through the InP:Fe is negligible, and thus the quality of the HVPE regrowth. At 10 °C, the maximum mean power is 254 mW, corresponding to a peak power of 11.5 W.

Furthermore, the evolution of the mean power with the duty cycle was measured, as represented in Fig. 1c. Our current source was limited to 26% of duty cycle, which was below the thermal roll-over both at 20 °C and 40 °C. It shows the heat load is efficiently dissipated through the laser top contact and the InP:Fe on its sides. At a duty cycle of 26%, the maximum mean power exceeds 1.6 W.

We measure the far-field by placing the power meter on an automated two-axis rotating stage. The scanning speed is around 0.6 degree per second. We use the same current source and average power meter as for the PIV experiments. A scan is performed with the laser turned off to suppress the thermal background. Both horizontal and vertical far-fields are shown in Fig. 1d. As for typical QCL, the vertical divergence is large, the full-width at half-maximum (FWHM) is 45.5° at 14 V, but is weakly depending on the operating point. However, the horizontal far-field remains single-lobed up to a 12 V bias and is only affected by a shoulder afterwards, whereas BA QCLs usually experience multi-lobed far-fields significantly degrading with increasing current. The measured horizontal FWHM is ranging from 11° to 13.1° from 11 V to 15 V applied voltage. As the bias is increasing, the peak horizontal emission is right-shifted from 0.5° to 3.2°. This beam steering is attributed to beating between the lowest order transverse modes, their effective refractive indices being close to each other. Generally speaking, it is induced by stable phase coherence through four-wave mixing interactions^24^ due to the larger nonlinear susceptibility in QCLs^25^.

**Figure 2:**
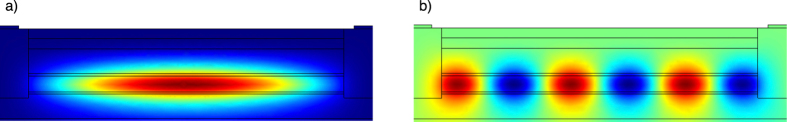
Simulated TM0 (**a**) and TM5 (**b**) electric field intensity.

In fact, the transverse optical modes that can exist in the cavity have been estimated by solving Maxwell’s equations using a 2D solver. For the simulation, the refractive indices are chosen to be 3.19 for the active region, 1 for the forming the passivation layer, 3.09 for the InP:Fe. The large optical cavities and the cladding layers are modeled by Drude model with a high frequency permittivity of *ε*_∞_ = 9.61, an effective electron mass to free electron mass ratio of 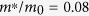 and an electron scattering time of *τ*_*scat*_ = 0.1 ps. Resulting fundamental and highest order modes, TM0 and TM5, are shown in Fig. 2. The overlaps of the modes with the active region and their calculated effective refractive index are summarized in Table 1.

The higher the mode order, the more it spills into the InP:Fe. In comparison, in the case of a standard double trench (DT) device, the overlap difference is lower between the modes as they are all strongly confined by the dielectric layer. In the case of the studied device, the overlap difference between TM0 and TM5 is ΔΓ = 3.1% whereas it is only ΔΓ_*DT*_ = 0.87% for a 32 μm DT device. Thus, the InP:Fe is acting as a high-order mode filter. In addition, the effective refractive index of TM0 is 3.1305, which is below the active region refractive index. Therefore, the refractive index variation is negative, and in accordance with the results in near-IR previously mentioned we expect to be able to change the energy distribution between the modes, in order to favor the fundamental one, by using optical feedback.

### Optical feedback experiments

The rate equation governing the complex electric field of the QCL subject to optical feedback is given by ref. 17:


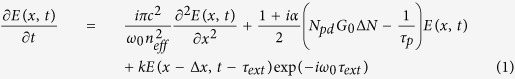


where *c* is the light velocity, *ω*_0_ the free-running angular frequency, *n*_*eff*_ the effective refractive index, *τ*_*p*_ the photon lifetime inside the laser cavity. *G*_0_ corresponds to the net modal gain for one period, *N*_*pd*_ to the number of periods, *α* to the linewidth enhancement factor and *τ*_*ext*_ to the external cavity roundtrip time. Δ*N* is the carrier density difference between the upper and the lower lasing states. Finally, *k* is the feedback coefficient, defined in the case of Fabry-Perot lasers as:


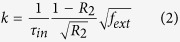


with *τ*_*in*_ the internal cavity roundtrip time, *R*_2_ the front facet reflectivity (here *R*_2_ = 0.3) and *f*_*ext*_ the feedback ratio, i.e. the ratio between reinjected and emitted light.

In BA lasers, the dependency of the field and carrier densities on the spatial variable *x* becomes very important, as underlined by the diffraction term in the complex field rate equation 1. A diffusion term furthermore exists in the carrier rate equation^26^. When applying optical feedback, the reinjected mode is not necessarily superimposed on the corresponding emitted mode, it can be shifted by a quantity Δ*x*. In this work, the influence of the spatial position of the reinjected beam will be studied, and the angle of the feedback mirror *θ* can therefore be adjusted in order to sweep the feedback over the whole active area, with a measurement precision estimated to ±2′. Furthermore, a shutter on the feedback path enables spatial filtering of the reinjection, by choosing which part of the beam is fed-back in the laser cavity.

**Figure 3:**
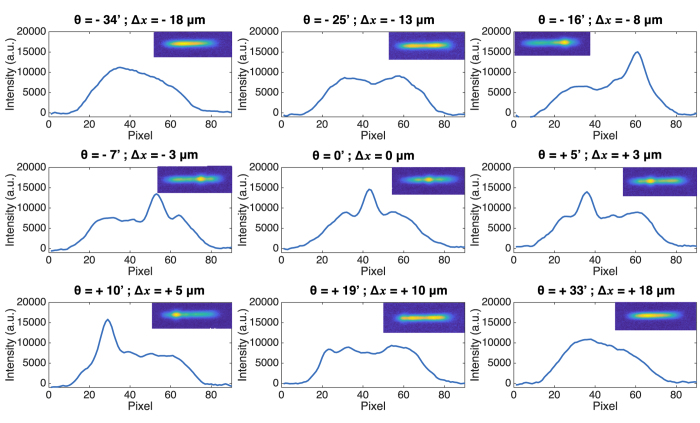
Near-field at the laser facet for different feedback angles, expressed both in arc-minute and in displacement on the laser facet with respect to the central position. The shutter is open and has no impact on the feedback. Figures in inset correspond to the near-field recorded on the camera.

Figure 3 presents the near-field profiles of the QCL previously described operated close to threshold (at 11.19 V with a 3% duty cycle) when subjected to conventional optical feedback. In each configuration, the profile is calculated by summing the intensities on each pixel column, and the inset presents directly the near-field recorded on the camera. The nine curves correspond to different feedback mirror angles, as indicated above the plots. The first and last curves are the free-running cases, where the reinjected beam does not enter the laser cavity. When changing the feedback angle, the impact of optical feedback is shown to be perfectly symmetrical with respect to the central position, corresponding to Δ*x* = 0.

Under free-running operation (*θ* = −34′ and +33′), the QCL near-field is not completely symmetric, there is more power on the left-hand side of the profile. This originates from beam-steering effect, which has been often observed in BA QCLs^27^. This beam-steering can be compensated by reinjecting a small amount of optical feedback on one side of the cavity. In that case, the power distribution becomes almost homogeneous over the near-field profile (*θ* = −25′ and +19′). Afterwards, when directing the feedback closer to the center of the cavity, a peak appears on the near-field profiles, corresponding to the position where the light is reinjected, and this peak is shifted continuously along the near-field when changing the mirror angle (from *θ* = −16′ to +10′). In particular, the transverse mode with three maxima TM2 becomes preponderant for centered optical feedback (*θ* = 0′). It is however important to stress that, although the power distribution between the several modes is strongly modified by optical feedback, the total emitted power is hardly affected, whatever the position of the reinjected beam.

**Figure 4:**
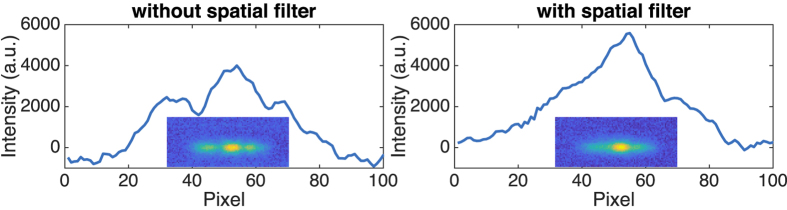
Near-fields at the laser facet with centered optical feedback and two shutter apertures. The shutter is fully open (**a**) and partially closed to filter the central lobe (**b**).

On this central position where the TM2 mode is predominant, a shutter is added on the feedback path close to the beam-splitter in order to spatially filter the central peak of the reinjected mode. As shown in Fig. 4, when the shutter is fully open, the TM2 mode appears, where the less pronounced peaks can be explained by a different contrast on the camera. Finally, when the shutter is partially closed to let through only the central peak, a spatial profile closer to the fundamental transverse mode TM0 is obtained. This is consistent with studies of optical feedback on BA laser diodes, that have shown that spatial filtering of the feedback leads to the excitation of lower order transverse modes^18^.

**Figure 5:**
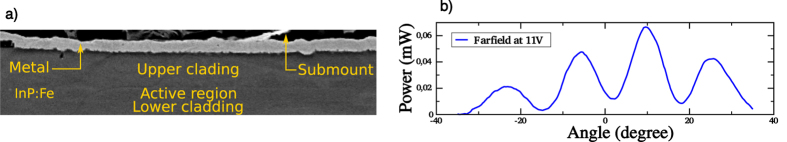
SEM picture of the laser with a cracked facet (**a**) and horizontal far-field at 11 V (**b**).

A second QCL is considered, which has the same design and same width than the first one. Although the power performances are similar, the horizontal far-field shows many lobes even at low bias voltage, as represented in Fig. 5b. These deteriorated performances can be understood by observing the facet, as shown in Fig. 5a. A crack is indeed observed on the right-hand side of the facet. This defect breaks the symmetry of the device, which leads to an inhomogeneous gain. Some nonlinear effects will be enhanced, such as spatial hole burning, which is responsible for the multi-lobe far-field.

**Figure 6:**
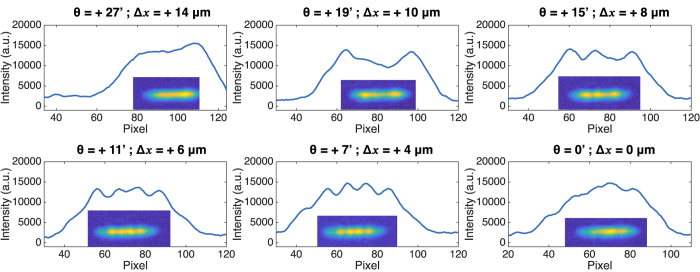
Near-field at the facet of the defective laser for different feedback angles. Only half of the angle excursion is shown, the other half presenting a symmetric behavior.

When subjecting this QCL to optical feedback, a response very different from the previous laser is observed, as depicted in Fig. 6. The near-field patterns are no longer symmetrical with respect to the centered feedback case, and here only the most interesting half of the way is shown, from the free-running to the case where Δ*x* = 0. When changing the feedback angle towards the center of the cavity, more and more transverse modes are excited. The succession of TM1 (*θ* = +19′), TM2 (*θ* = +15′), TM3 (*θ* = +11′) and TM4 *θ* =+5′ are observed. Finally, for centered optical feedback, TM5 is excited (*θ* = 0′), although the extinction between the lobes is not very clear. These observations are consistent with the conducted simulations showing that a maximum of six transverse modes can co-exist in the 32 μm cavity. The appearance of these consecutive TM modes is probably due to multi-path interference^28^, leading to multiple overlaps between the different modes in the active region and the delayed field depending on the mirror tilt.

Furthermore, these near-field patterns where higher order transverse modes appear in the case of optical feedback resemble the situation described in BA laser diode very sensitive to spatial hole burning^17^. This might suggest the appearance of filamentation in this BA QCL presenting a defect on the facet, although a temporal study would be necessary in order to conclude on this point. Similar field distributions and response to optical feedback should be expected in the case of any BA QCL presenting an asymmetry or an inhomogeneous gain, leading to a multi-lobe far-fied.

**Figure 7:**
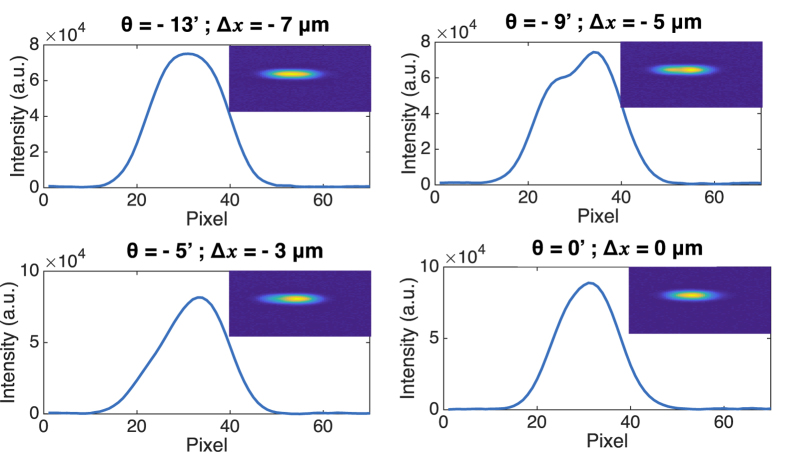
Near-field at the laser facet for the 14 μm-wide laser for different feedback angles. Only half of the angle excursion is shown, the other half presenting a symetric behavior.

In order to evaluate the impact of the QCL width on its response to optical feedback, an additional measurement was performed on a 14 μm-wide QCL. This laser has the same active region design as the one described previously, and was processed using a standard DT technique. According to the simulations, three modes can exist in this cavity, but the beam profile of the free-running laser is gaussian, as shown in the first plot of Fig. 7. This QCL can no longer be considered as a BA laser, and its response to centered optical feedback is indeed the one of a narrow-ridge laser, with an increase of the output power and a narrowing of the near-field profile.

However, when rotating the feedback angle, the higher order mode TM1 can be excited (*θ* = −9′ and −5′), as depicted in Fig. 7. We observe the same tendency as in the case of BA QCL under feedback, with a limited displacement of the feedback peak due to the smaller width of the cavity and the limited number of modes that can get excited. Therefore, this study on a 14 μm QCL can be considered as the limit case where the spatial dimension *x* of optical feedback must be taken into account.

In summary, the response of a BA QCL to optical feedback is evidenced for the first time. The 32 μm-wide QCL under study presents high performances owing to the InP:Fe grown on both sides of the ridge. In particular, the laser exhibits high output power, efficient heat-load dissipation and the controlled optical feedback substantially purifies the beam quality.

This study shows the possibility to engineer the emission pattern of BA QCLs using a nonlinear external control with and without spatial filtering. Tailoring the near-field emission pattern becomes possible, even with a small amount of off-centered reinjected light which is a non-monolithic solution, much easier to implement. It can be changed either to a more homogeneous distribution or to a near-field that presents an intensity peak following the mirror displacement for a QCL emitting on a single lobe. Spatially-filtered optical feedback is used to further enhance the near-field profile quality. In the case of a BA QCL with a multi-lobe far-field, all the cavity transverse modes are sequentially excited while changing the feedback mirror angle. The comparison with a narrower device points out the impact of the number of cavity modes on the feedback response.

Further works will investigate the dynamical behavior of QCLs under optical feedback. As a matter of fact, depending on the QCL structure quality, strong spatial hole burning can arise, which could lead to filamentation. Optical feedback can be used as an efficient solution to stabilize the QCL dynamics, as for BA near-infrared laser diodes. Furthermore, the design of the spatial filters should be optimized to reach a better control of the reinjected beam, and therefore on the device far-field pattern. In addition, further experiments will be performed in order to explore the impact the optical feedback has on BA devices of several hundreds of microns, typically from 100 to 500 μm.

## Methods

### Design and processing

The QCL active region design is derived from the shallow well structure previously published^23^, and adapted to have a gain centered around 4.6 μm. A 2.5 μm thick n-doped InP layer (*n* = 10^17^ cm^−3^), acting as the bottom optical cladding layer, is grown by MBE, followed by a 200 nm Ga_0.47_In_0.53_As layer (*n* = 6 × 10^16^ cm^−3^), which plays the role of a large optical cavity (LOC). Finally, we grow the 30 period active region and another similar LOC.

Right after the MBE growth, the ridge is defined with a SiO_2_ hardmask and standard lithography process, and we process it by Cl_2_ -based ICP etching. After photoresist removal, InP:Fe is regrown by low pressure HVPE on the sides of the ridges. Then, SiO_2_ the hardmask is removed and the upper cladding layers are grown by MOVPE. It is composed by two InP layers and a Ga_0.47_In_0.53_As contact layer, of 2.7 μm (*n* = 10^17^ cm^−3^), 1 μm (*n* = 1.5 × 10^19^ cm^−3^) and 1 μm (*n* = 2 × 10^19^ cm^−3^), respectively.

Finally, the device is passivated with SiO_2_, a Ti/Pt/Au top contact and a 5 μm -thick gold pad are deposited, the substrate is thinned down to 150 μm and a Ti/Pt/Au bottom contact is realized.

### PIV curves measurements

In order to measure the PIV curves, the laser submount is set on a copper heat sink which temperature is controlled with a Peltier cooler. The duty cycle is 3%, the pulses lasting 600 ns. The mean power is measured behind an aspherical lens (f = 1.87 mm, NA = 0.87). The collection factor was evaluated to be 0.79 by comparing the maximum optical power with this setup with the one read from a second power meter with high aperture angle placed right after the laser facet.

The current drawn by the BA QCL at maximum power is about 5.5 A. For such high currents, the pulse rise time is no longer negligible and the peak power is evaluated from the time integral of the light pulse, measured with a fast mercury-cadmium-telluride photodetector, from the equation:


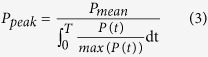


where *T* is the period of the pulses, *P(t*) is the power read on the oscilloscope and *P*_*mean*_ is the mean power.

### Experimental setup for feedback experiments

**Figure 8:**
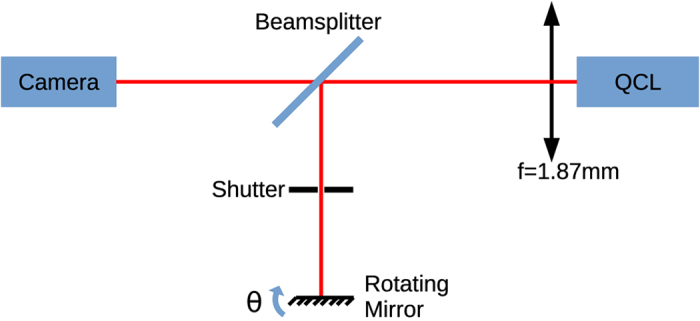
Experimental setup. The mirror is mounted on a precision rotation stage with vernier scale to control the angle of feedback.

In order to characterize the QCL behavior under external optical feedback, we consider the experimental setup described in Fig. 8. The emitted light is collected at the output of the laser and split into a feedback path and a detection path using a 60/40 beam splitter. On the feedback path, part of the light is reinjected into the laser after reflection on a rotating mirror. On the detection path, the very short focal length *f* = 1.87 mm of the lens enables imaging the near-field of the QCL on a camera, comporting 124 × 124 pixels. Since the camera is at the same distance from the laser facet as the feedback mirror, the beam is focused on the camera and on the mirror at the same time, and what is reinjected into the QCL is an image of its near-field. The external cavity length is chosen to be *L*_*ext*_ = 29 cm, but longer cavities were also considered and led to similar results, as long as the laser beam remains focused on the feedback mirror.

With the described experimental setup, it is not possible to measure exactly this feedback ratio. However, the observed threshold fluctuations of less than ±1% suggest that only a small amount of light is reinjected into the cavity, corresponding to feedback ratios of less than 5%. This small quantity of optical feedback should however lead to an improvement of the beam profile, whereas higher amount of reinjection would tend to destabilize the laser^16^.

## Additional Information

**How to cite this article**: Ferré, S. *et al*. Beam shaping in high-power broad-area quantum cascade lasers using optical feedback. *Sci. Rep.* **7**, 44284; doi: 10.1038/srep44284 (2017).

**Publisher's note:** Springer Nature remains neutral with regard to jurisdictional claims in published maps and institutional affiliations.

## Figures and Tables

**Table 1:** Overlap of the optical mode with the active region and effective refractive indices for the 6 existing transverse modes.

Mode order	Overlap with theactive region (%)	Effective refractive index
TM0	58.58	3.1305
TM1	58.38	3.1284
TM2	58.03	3.1248
TM3	57.51	3.1199
TM4	56.73	3.1136
TM5	55.51	3.1059
